# The Drug Clerk Law

**Published:** 1871-05

**Authors:** 


### The Drug Clerk Law.

Dr. Henry Saunders, Act. Ass't Surgeon, U. S. A., objects to the New Drug Clerk Law:—

1st, That its operation is confined to the CITY of New York, leaving all other cities, towns and villages in the State unprotected.

2d. That *owners* or *proprietors* of stores are not amenable to the law; only clerks!

If strictly enforced it will in time, drive all the incompetent clerks out of New York city into other parts of the State, or other States; and the question presents itself, "Why should the ctty of New York be favored thus at the expense of the whole State?" As regards the second defect, it is a fact well known to most physicians, that many persons more or less ignorant of chemistry and phaemacy are engaged in the drug business as owners of stores who rely almost entirely on the knowledge and ability of their clerks, but do not hesitate, in their absence, to sell drugs, dispense medicines, and compound prescriptions. And the question is why should *they* be exempted from the operation of this law.

